# *β*-composite Interval Mapping for robust QTL analysis

**DOI:** 10.1371/journal.pone.0208234

**Published:** 2018-12-03

**Authors:** Md. Mamun Monir, Mita Khatun, Md. Nurul Haque Mollah

**Affiliations:** 1 Department of Statistics, University of Rajshahi, Rajshahi, Bangladesh; 2 Institute of Bioinformatics, Zhejiang University, Hangzhou, China; College of Agricultural Sciences, UNITED STATES

## Abstract

Interval mapping approaches have been playing significant role for quantitative trait locus (QTL) mapping to discover genetic architecture of diseases or traits with molecular markers. Composite interval mapping (CIM) is one of the superior approaches of the interval mapping for discovering both linked and unlinked putative QTL positions. However, estimators of this approach are not robust against phenotypic outliers. As a result, it fails to detect true QTL positions in presence of outliers. In this study, we investigated the performance of *β*-Composite Interval Mapping (BetaCIM) for detecting both linked and unlinked important QTLs positions from the robustness points of views. Performance of this approach depends on the value of tuning parameter *β*. It reduces to the classical CIM approach for *β* →0. We described and formulated the cross-validation procedure for selecting trait specific optimum value of *β*. It was observed that the optimum value of *β* depends on both amount of contaminated observations and their scatteredness. BetaCIM approach discover similar QTL positions as classical IM/CIM in absence of phenotypic outliers, but gives better results in presence of phenotypic outliers in terms of detecting true QTLs and effects estimation. We formulated the generalized forms of robust QTL analysis and developed an R-package named “BetaCIM” by implementing this robust approach. Left and right kidney weight data sets of mouse intercross population (129 S1/SvlmJ × A/J) were analyzed by using BetaCIM, CIM, and IM approaches. For right kidney weight (RKW) CIM and BetaCIM provided similar LOD score profile, and both approaches identified 3 QTL positions. IM approach also identified 3 QTL positions. For left kidney weight (LKW), there was evidence of one outlying observation; and in this case the BetaCIM approach identified 2 QTL positions. However, none of the QTLs were significant by CIM and IM approaches at 5% level of significance. Gene expression ontology (GEO) search showed that the candidate genes (*Otof* and *A330033J07Rik*) of the identified QTLs for LKW were expressed in kidney. Both simulation and real data analysis results showed that BetaCIM approach improves the performance over the existing methods in presence of phenotypic outliers. Otherwise, it keeps almost equal performance.

## Introduction

QTL mapping approaches have been used successfully to discover the genetic variants by using experimental cross populations [1, 2]. Arranging a cross between two inbred lines, which are different in quantitative trait, then scoring the segregating progeny for the trait and for a number of genetic marker are the basic steps of QTL mapping [3]. Due to advances in molecular biology, the availability of genetic markers has rapidly increased, leading to extensive use of QTL mapping in genetic studies of quantitative traits.

Genetic effects associated with marker genotypes are generally confounded by the position of functional QTL and its actual effect. By exploiting this property, interval mapping [3] has become a superior way to find QTLs [4, 5]. However, in the case of multiple linked QTLs, genetic effects need to be sufficiently separated [6]. Interval-mapping approach cannot separate the effects of linked putative positions completely [7]. Composite Interval Mapping (CIM) is a powerful analytical technique that improves the reliability and accuracy of QTL mapping by separating the effect of QTL from its locations [6, 8]. The CIM approach has advantages over other mapping methods for detecting linked QTLs. By utilizing the properties of multiple regression, test statistic of this approach was constructed to be unaffected by the QTLs outside of testing interval [6]. CIM can eliminate influence of genetic background by using a set of representative markers of the background QTL as covariates. Likelihood ratio test statistics for this approach was constructed by joining interval mapping for QTL position and multiple regression analysis for background markers that permits to identify linked QTL located near at the same chromosome.

Real phenotypic data might be contaminated by some abnormal observations, known as outliers. In general, outliers have a large impact on any classic statistical estimator. For QTL mapping, the presence of phenotypic outlying observations might be the reason for misrepresenting multiple linked QTLs and could hinder the efficient and accurate resolvability of QTLs [9]. For experimental population, phenotypic datasets often contain outlying observations that may seriously affect the estimates of model parameters and can lead to the wrong detection of QTL positions and their estimated effects [10, 11]. Mollah and Eguchi [12, 13] proposed *β*-Composite Interval Mapping (BetaCIM) for robustly identifying putative QTL positions, where parameters were estimated by maximizing *β*-likelihood functions. Maximization of *β*-likelihood function is equivalent to minimization of Beta-Divergence [14–17]. However, in their study, they did not provide any theoretical discussion about the robustness. Moreover, they did not investigate the performance for detection of linked QTLs. In this work, we conducted simulation study to observe the performance of BetaCIM approach for detecting multiple linked and unlinked QTLs and compared the analysis results with classical interval mapping (IM) and composite interval mapping (CIM) approaches in presence and absence of phenotypic outliers. We provided the theoretical discussion about the robustness property, cross validation procedure of selecting the tuning parameter *β*, and generalized the formulas of BetaCIM approach. Two real datasets, left and right kidney weight data sets of mouse intercross population (129 S1/SvlmJ × A/J), were analyzed to demonstrate the usefulness of using BetaCIM approach for QTL mapping. We developed an R package, named BetaCIM, for implementing this approach.

## Materials and methods

### Genetic model

Genetic cross between two parental inbred lines *P*_1_ and *P*_2_ is performed to produce an *F*_1_ population, consist of all heterozygotes genotypes, which are used to produce the segregating progeny *B*_1_ = *F*_1_× parent (backcross) or an *F*_2_ = *F*_1_×*F*_1_ (intercross). The genetic model for intercross population is as follows

$$G=\left[\begin{array}{c} G_{2}\\ G_{1}\\ G_{0} \end{array}\right]=\left[\begin{array}{c} 1\\ 1\\ 1 \end{array}\right]\mu +\left[\begin{array}{cc} 1 & -1/2\\ 0 & 1/2\\ -1 & -1/2 \end{array}\right]\left[\begin{array}{l} a\\ d \end{array}\right]=1_{3\times 1}\mu +DE$$ (1)

Here *G*_2_, *G*_1_ and *G*_0_ are the genotypic values of genotypes *QQ*, *Qq* and *qq*. ***D*** is known as the genetic design matrix and ***E*** = [*a*, *d*]^*T*^ is the vector of genetic parameters. The first and second columns of ***D***, denoted by *D*_1_ and *D*_2_, represent the status of the additive and dominance effects.

The genetic model of backcross population can be written as

$$G=\left[\begin{array}{l} G_{2}\\ G_{1} \end{array}\right]=\left[\begin{array}{l} 1\\ 1 \end{array}\right]\mu +\left[\begin{array}{l} 1\\ 0 \end{array}\right]\left[a\right]=1_{2\times 1}\mu +DE$$ (2)

All notations are similar as described in the upper of this section.

### CIM statistical model for QTL mapping

Phenotypic traits might be controlled by several QTLs. Estimators of simple interval mapping approach might be biased for confounding effects of multiple background QTLs [18]. Usually QTLs are highly linkage disequilibrium with corresponding flanking markers, therefore highly significant markers choosing through stepwise procedure could be a good representative set of background QTLs. Composite interval mapping approach modified the simple interval-mapping approach by including several significant markers as cofactors. Some others confounding factors (e.g. sex, age, diet etc.) also might have influence on traits, can be used as cofactor in the model to adjust their effects.

Suppose, we want to test for a QTL on a marker interval for *F*_2_ population, then the statistical model for composite interval mapping can be written as

$$y_{j}=ax_{j}^{*}+dz_{j}^{*}+X_{j}\gamma +\varepsilon_{j}$$ (3)

where *y*_*j*_ is the phenotypic value of the *j*^th^ individual; *a* is the additive effect of the testing QTL position; *d* is the dominance effect of testing QTL position; *X*_*j*_ is the matrix, may contain some chosen markers and other explanatory variables; *γ* is the vector of partial regression coefficients including the general mean effect *μ*; *ε*_*j*_ is a random error. The value of ($x_{j}^{*}$, $z_{j}^{*}$) is (1, -0.5) if QTL genotype is *QQ*, (0, 0.5) if genotype is *Qq*, and (-1, -0.5) if genotype is *qq*.

Statistical genetic model for backcross population can be written as

$$y_{j}=ax_{j}^{*}+X_{j}\gamma +\varepsilon_{j}$$ (4)

Notations of this equation are similar as described in upper of this section. The value of $x_{j}^{*}$ is 1 if QTL genotype is *QQ*, and 0 if genotype is *Qq*.

### Robustification of CIM approach using beta-likelihood estimators

Composite interval mapping approach use the estimators derived from classical likelihood function to estimate genetic parameters, which might produce false positive or reduce detecting power of true loci in presence of phenotypic outliers. Instead of classical estimators of genetic parameters, *β*-Composite Interval Mapping uses the robust estimators derived from beta-likelihood function for robustly estimating the QTL positions in presence and absence of phenotypic outliers.

In the model (3), observation *y*_*j*_’s are influenced by three QTL genotypes *QQ*, *Qq*, and *qq*; therefore, each phenotypic observation (*y*_*j*_) is assumed to follow a mixture of three possible gaussian densities with different means and mixing proportion. The distribution function of each phenotypic observation (*y*_*j*_’s) can be defined as

$$f(y_{j}|\theta ,X_{j})=\frac{1}{\sigma }\sum_{i=1}^{3}p_{ij}\varphi \left(\frac{y_{j}-\mu_{ji}}{\sigma }\right)$$

where *θ* = (*p*,*a*,*d*,*γ*,*σ*^2^), *ϕ*() is a standard normal probability density function, $\mu_{j1}=a-\frac{d}{2}+X_{j}\gamma$, $\mu_{j2}=\frac{d}{2}+X_{j}\gamma$, and $\mu_{j3}=-a-\frac{d}{2}+X_{j}\gamma$. The mixing proportions *p*_*ji*_’s, which are functions of the QTL position parameter *p*, are the conditional probabilities of QTL genotypes given marker genotypes. For *n* individuals, the objective function for estimating *θ* is defined as

$$L_{\beta }\left(\theta |Y,X\right)=\frac{1}{\beta }\left[\frac{1}{nl_{\beta }(\theta |X)}\sum_{j==1}^{n}{\left\{f(y_{j}|\theta ,X_{j})\right\}}^{\beta }-1\right]$$ (5)

where *l*_*β*_(*θ*|*X*) = [∫{*f*(*y*|*θ*,*X*}^*β*+1^*dy*]^*β*/(*β*+1)^

It was induced from the beta-divergence [19] for estimation of the parameters. It reduces to the log likelihood function for *β* →0. That is

$$\underset{\beta \to 0}{\lim}L_{\beta }(\theta |Y,X)=L_{0}(\theta |Y,X)$$

Therefore, the objective function (5) called as beta-likelihood function. Maximization of this type of beta-likelihood function is equivalent to the minimization of beta-divergence [19] for estimating model parameter *θ*. The *β-*LOD score for the evidence of a QTL in a marker interval from the robustness point of view is defined by

$$LOD_{\beta }=0.434n\left\{\underset{\theta }{\sup}L_{\beta }\left(\theta |Y,X\right)-\underset{\theta_{0}}{\sup}L_{\beta }\left(\theta |Y,X\right)\right\}$$ (6)

where *θ*_*0*_ and *θ* are the restricted and unrestricted parameter spaces. For *β* →0, the LOD_*β*_ reduces to the classical LOD criterion. The threshold value to reject the null hypothesis can be computed by permutation test [19].

### Generalized form of the formulas for QTL mapping

The formulas for robust estimators of genetic parameters were elaborately described in related publications [12, 13]. In this section, we described the generalized forms of the robust estimators for QTL mapping. If *k* marker intervals (*k* putative QTLs) are considered jointly in mapping, the dimension of the genetic design matrix ***D*** augment to 2^*k*^ × *k* for a backcross population and to 3^*k*^ × 2*k* for an *F*_2_ population when epistasis is ignored. Let us consider a backcross population as an example to see how to use these general formulas for other genetic models and populations. If we want to consider three marker intervals (three putative QTLs) simultaneously and use an additive model, the genetic model can be defined as

$$G=\left[\begin{array}{l} G_{111}\\ G_{110}\\ G_{101}\\ G_{100}\\ G_{011}\\ G_{010}\\ G_{001}\\ G_{000} \end{array}\right]=\left[\begin{array}{l} 1\\ 1\\ 1\\ 1\\ 1\\ 1\\ 1\\ 1 \end{array}\right]\mu +\left[\begin{array}{ccc} 1/2 & 1/2 & 1/2\\ 1/2 & 1/2 & -1/2\\ 1/2 & -1/2 & 1/2\\ 1/2 & -1/2 & -1/2\\ -1/2 & 1/2 & 1/2\\ -1/2 & 1/2 & -1/2\\ -1/2 & -1/2 & 1/2\\ -1/2 & -1/2 & -1/2 \end{array}\right]\left[\begin{array}{c} a_{1}\\ a_{2}\\ a_{3} \end{array}\right]=1_{8\times 1}\mu +DE$$ (7)

where *G*_111_, *G*_110_, *G*_101_, *G*_100_, *G*_011_, *G*_010_, *G*_001_ and *G*_000_ represent genotypic values of QTL genotypes *AABBCC*, *AABBCc*, *AABbCC*, *AABbCC*, *AABbCc*, *AaBBCC*, *AaBBCc*, *AaBbCC* and *AaBbCc* respectively. The notations *a*_1_, *a*_2_ and *a*_3_ represent the effects of QTLs A, B, and C respectively. The genetic design matrix ***D*** with dimension 8 × 3 specifies that the corresponding likelihood is a mixture of 8 normal densities and has 3 genetic parameters (excluding *μ*) to be estimate. Accordingly, the matrix of *β* weighted posterior probabilities Π_*β*,_ defined in the related publication [12], is an n × 8 matrix. In deriving the *β*-estimators, in the E-step Π_*β*_ of the eight QTL genotypes are updated, and in the M-step the following equations

$$E^{\left(t+1\right)}=m^{\left(t\right)}-M^{\left(t\right)}E^{\left(t\right)}$$ (8)

$$\gamma^{\left(t+1\right)}={\left[X^{T}\;\{X\;\#(\Pi_{\beta }^{\left(t\right)}1)\}\right]}^{-1}\left[X^{T}\;\left\{Y\;\#\left(\Pi_{\beta }^{\left(t\right)}1\right)-\Pi_{\beta }^{\left(t\right)}DE^{\left(t+1\right)}\right\}\right]$$ (9)

$$\begin{array}{l} \sigma^{2(t+1)}=(1+\beta )[{\left(Y-X\gamma^{\left(t+1\right)}\right)}^{T}\{(Y-X\gamma^{\left(t+1\right)})\#(\Pi_{\beta }1)\}\\ \;-2{\left(Y-X\gamma^{\left(t+1\right)}\right)}^{T}\Pi_{\beta }^{\left(t\right)}DE^{\left(t+1\right)}-E^{T(t+1)}V^{\left(t\right)}E^{\left(t+1\right)}]\\ \;{\left[1^{T}\Pi_{\beta }^{\left(t\right)}1\right]}^{-1} \end{array}$$ (10)

are applied to maximize *Q*(θ | θ^(t)^). Here,

$$V=\left[\begin{array}{ccc} 1^{T}\Pi_{\beta }(D_{1}\#D_{1}) & 1^{T}\Pi_{\beta }(D_{1}\#D_{2}) & 1^{T}\Pi_{\beta }(D_{1}\#D_{3})\\ 1^{T}\Pi_{\beta }(D_{2}\#D_{1}) & 1^{T}\Pi_{\beta }(D_{2}\#D_{2}) & 1^{T}\Pi_{\beta }(D_{2}\#D_{3})\\ 1^{T}\Pi_{\beta }(D_{3}\#D_{1}) & 1^{T}\Pi_{\beta }(D_{3}\#D_{2}) & 1^{T}\Pi_{\beta }(D_{3}\#D_{3}) \end{array}\right],$$

$$m=\left\{\frac{{\left(Y-X\gamma \right)}^{T}\Pi_{\beta }D_{i}}{1^{T}\Pi_{\beta }(D_{i}\#D_{i})}\right\}\;and\;M={\left\{\frac{1^{T}\Pi_{\beta }(D_{i}\#D_{j})}{1^{T}\Pi_{\beta }(D_{i}\#D_{i})}\times \delta (i\neq j)\right\}}_{3\times 3}.$$

*δ* is an indicator variable. For details about *Q*(θ | θ^(t)^) and Π_*β*,_ see the related publication [12]. To infer the joint conditional probability matrix ***Q*** for the three putative QTLs, we use the property that if there is no interference in crossing over, the conditional distributions of the individual putative QTL genotypes given the flanking marker genotypes are independent, irrespective of whether the QTLs are linked or not. This independence property simplifies the inference of ***Q*** matrix. If pair-wise epistasis of QTLs A × B, A × C, and B × C are also analyzed, the dimensions of genetic design matrix ***D*** in Eq (7) augment to 8 × 6. Columns 4, 5, and 6, which are the products of columns 1 and 2, 1 and 3, and 2 and 3, of the genetic design matrix represents the status of the epistatic parameters of different genotypes. The ***Q*** matrix is the same as that for the additive model. If higher order of QTL epistasis is considered, the corresponding column vector of ***D*** can be extended by the same procedure. For more details discussion about general formulas for CIM algorithm, please see the paper of Kao and Zeng [20].

### Robustness

Let *G* be the distribution function of *g*, then we can view the *β*-estimator, which is a function of *G* defined by

$$\begin{array}{l} \theta_{\beta }[G]=\underset{\theta }{\arg\;\min}\;D_{\beta }\left(g,f_{\theta }\right)\\ \;=\underset{\theta }{\arg\;\max}\;\left\{\int \Omega_{\beta }\left(y;\theta \right)dG\left(y\right)\right\} \end{array}$$ (11)

where

$$\Omega_{\beta }\left(y\right)=\frac{1}{\beta C_{\beta }\left(\theta \right)}{\sum_{i=1}^{3}\left[\frac{p_{i}}{\sigma }\phi \left(\frac{y-\mu_{i}}{\sigma }\right)\right]}^{\beta }\times \pi_{i}^{\left(t\right)}-\frac{1}{\beta }$$ (12)

is the objective function. Therefore, the robustness of the *β*-estimator can be investigated by the influence function. The influence function (IF) for the *β*-estimator at *y* under the distribution function *G* is defined as

$$IF(y;\theta_{\beta },G)=\underset{\varepsilon \to 0}{\lim}\left\{\theta_{\beta }\left[\left(1-\varepsilon \right)G+\varepsilon \Delta_{y}\right]-\theta_{\beta }\left[G\right]\right\}/\varepsilon$$ (13)

where Δ_*y*_ is the probability measure that puts mass 1 at the point *y*. An estimator is said to be B-robust if its influence function is a bounded function of *y* [21]. Since the *β*-estimator satisfies the properties of M-estimator, the influence function for the robust estimator also can be written as

$$IF\left(y;\theta_{\beta },G\right)=H{\left(\psi_{\beta },G\right)}^{-1}\psi_{\beta }\left(y;\theta_{\beta }\left[G\right]\right)$$ (14)

where *ψ*_*β*_(*y*;*θ*) = ∂Ω_*β*_(*y*;*θ*)/∂*θ* is the estimating function for the *β*-estimator and

$$H\left(\psi_{\beta },G\right)=-{\int \left[\frac{\partial \psi_{\beta }(y;\theta )}{\partial \theta }\right]}_{\theta =\theta_{\beta }[G]}dG\left(y\right)$$ (15)

is a matrix which does not depend on *y*; thus, the B-robustness is equivalent to the boundness of the estimating function for the M-estimator as well as the *β*-estimator [14, 22]. To prove the boundedness of the estimating function *ψ*_*β*_(*y*;*θ*) for the *β*-estimator, let us consider the general form of estimating function as defined by

$$\begin{array}{l} \psi_{\beta }\left(y;\theta \right)=\frac{1}{\beta }\sum_{i=1}^{3}{\left[\frac{p_{i}}{\sigma }\phi \left(\frac{y-\mu_{i}}{\sigma }\right)\right]}^{\beta }\times \pi_{i}^{\left(t\right)}\frac{\partial {\left[C_{\beta }\left(\theta \right)\right]}^{-1}}{\partial \theta }+\\ \;{\left[C_{\beta }\left(\theta \right)\right]}^{-1}\sum_{i=1}^{3}{\left[\frac{p_{i}}{\sigma }\phi \left(\frac{y-\mu_{i}}{\sigma }\right)\right]}^{\beta }\frac{\partial \log\left[\frac{p_{i}}{\sigma }\phi \left(\frac{y-\mu_{i}}{\sigma }\right)\right]}{\partial \theta }\times \pi_{i}^{\left(t\right)} \end{array}$$ (16)

Obviously, the boundedness of the estimating function depends only on the second term of the right-hand side of (16), since *C*_*β*_(*θ*) is independent on observations. In the second term of the right-hand side of (16), we have

$$\frac{\partial \log\left[\frac{p_{i}}{\sigma }\phi \left(\frac{y-\mu_{i}}{\sigma }\right)\right]}{\partial a}=\left\{ \begin{array}{l} +(y-\mu_{1})/\sigma^{2},\;for\;i=1\\ 0,\;for\;i=2\\ -(y-\mu_{3})/\sigma^{2},\;for\;i=3 \end{array}\right.$$ (17)

$$\frac{\partial \log\left[\frac{p_{i}}{\sigma }\phi \left(\frac{y-\mu_{i}}{\sigma }\right)\right]}{\partial d}=\left\{ \begin{array}{l} -(y-\mu_{1})/2\sigma^{2},\;for\;i=1\\ +(y-\mu_{2})/2\sigma^{2},\;for\;i=2\\ -(y-\mu_{3})/2\sigma^{2},\;for\;i=3 \end{array}\right.$$ (18)

$$\frac{\partial \log\left[\frac{p_{i}}{\sigma }\phi \left(\frac{y-\mu_{i}}{\sigma }\right)\right]}{\partial \gamma }=\left(y-\mu_{i}\right)X^{T}/\sigma^{2}$$ (19)

$$\frac{\partial \log\left[\frac{p_{i}}{\sigma }\phi \left(\frac{y-\mu_{i}}{\sigma }\right)\right]}{\partial \sigma^{2}}=\left[{\left(y-\mu_{i}\right)}^{2}-\sigma^{2}\right]/2\sigma^{4}$$ (20)

Thus, we can conclude that if *β* > 0, then all components of estimating function are bounded with respect to *y* and *X*. This is because all the terms corresponding to the Eq (17–20) are of the form $e^{-\beta z^{2}}f\left(z\right)$ with *f*(*z*) being polynomial in *z*, which is bounded in z∈ℜ. In case of *β* = 0 or equivalently the maximum likelihood estimator, all the terms corresponding to the Eq (17–20) become unbounded. Typically, for example, $\sup_{z}\left|e^{-\beta z^{2}}z\right|=e^{-\frac{1}{2\beta }}/\sqrt{2\beta }$. Thus, we may conclude that the estimating function *ψ*_*β*_(*z*;*θ*) for the *β*-estimator is bounded for *β* > 0. Therefore, the *β*-estimators are B-robust against outliers.

### Selection of the tuning parameter *β*

The value of the tuning parameter *β* plays a key role in the performance of the BetaCIM method. It controls the trade-off between robustness and efficiency of estimators. This method shows good performance for a wide range of *β*. A large *β* decreases the efficiency and increases the robustness of an estimator, and vice-versa for the smaller *β*. However, an optimum value for *β* depends on the initialization of model parameters, data contamination rates, type of data contamination, type of datasets and so on. So heuristic selection of the tuning parameter *β*, may produces misleading results in some satiations. To find an optimum *β* for minimum *β*-divergence method, Mollah et al. [16, 17] used *β*-divergence with a fixed value *β*_0_ of *β* as a measure for evaluation of the minimum *β*-divergence estimators. In this paper, we also use the same measure for *β* selection using cross validation. To define the measure for *β* selection using *K*-fold cross validation, the entire dataset $D=\{(y_{j},X_{j}):j=1,2,\ldots ,n\}$ into *K* subsets $D_{1}$, $D_{2}$, …, $D_{k}$ and let $D_{k}^{\prime }=\{(y_{j},X_{j}):(y_{j},X_{j})\notin D_{k}\}$. Then the measure for *β* selection by *K*-fold cross validation can be defined by

$$D_{\beta_{0}}(\beta )=\frac{1}{n}\sum_{k=1}^{K}L_{\beta_{0}}^{\prime }({\hat{\theta }}_{\beta }|D_{k})$$ (21)

where ${\hat{\theta }}_{\beta }=({\hat{a}}_{\beta },{\hat{d}}_{\beta },{\hat{\gamma }}_{\beta },{\hat{\sigma }}_{\beta }^{2})$ are estimated using dataset $D_{k}^{\prime }$ and

$$L_{\beta_{0}}^{\prime }({\hat{\theta }}_{\beta }|D_{k})=\frac{1}{\beta_{0}}\left[1-\frac{1}{n_{k}l_{\beta_{0}}({\hat{\theta }}_{\beta }|X)}\sum_{(y,X)\in D_{k}}{\left\{f(y|{\hat{\theta }}_{\beta },X)\right\}}^{\beta_{0}}\right]$$ (22)

with

$$l_{\beta_{0}}({\hat{\theta }}_{\beta }|X)={\left[\int {\left\{f(y|{\hat{\theta }}_{\beta },X\right\}}^{\beta_{0}+1}dy\right]}^{\beta_{0}/(\beta_{0}+1)}$$ (23)

where *n*_*k*_ is the number of observation in the subset $D_{k}$. Under null hypothesis the distribution function is $f(y|{\hat{\theta }}_{\beta },X)=\frac{1}{{\hat{\sigma }}_{\beta }}\phi \left(\frac{y-X{\hat{\gamma }}_{\beta }}{{\hat{\sigma }}_{\beta }}\right)$, where ${\hat{\theta }}_{\beta }=({\hat{\gamma }}_{\beta },{\hat{\sigma }}_{\beta }^{2})$. We select an appropriate *β* by the minimizer of $D_{\beta_{0}}(\beta )$ for *β*. To compute *β*-LOD score for testing the evidence of a QTL in a specific position in a chromosome, we need to select *β* by cross validation under null hypothesis and alternative hypothesis separately. So, it takes a lot of time to compute the genome-wide *β*-LOD scores. However, we can use the same *β* by cross validation under null hypothesis, for each of alternative cases also to save the computational time, since the estimators show good performance for a wide range of *β* > 0 also. If cross validation results find the value of *β* significantly larger than 0, it indicates that outliers contaminate the dataset. If cross validation results find the value of *β* very close to 0, it indicates that the dataset is not contaminated by outliers and the robustified CIM algorithm reduce to the traditional CIM.

## Results

### Simulation study

Simulations studies were conducted for Backcross (*BC*) and Intercross (*F*_2_) populations based on the assumptions of multiple linked and unlinked QTLs, and in presence and absence of phenotypic outliers. LOD scores were calculated for classical interval mapping and composite interval mapping approaches and *β*-LOD scores were calculated for BetaCIM approach that is equivalent to classical LOD scores for *β*→0. Trait specific optimum *β* was selected by using *k*-fold cross-validation procedure, implemented in our BetaCIM R-package. We simulated genotype data by using the implemented functions (sim.map and sim.cross) of popular R/qtl package. Then by setting effects corresponding to some specific markers the phenotypic data sets were generated. Mean sum of squares of the parameters were calculated by using the following formula-

$$MSE=\frac{1}{p}\sum_{i=1}^{p}{\left(\theta_{i}-{\hat{\theta }}_{i}\right)}^{2}.$$

Detection power of the QTL was calculated as the numbers of times calculated LOD scores of a QTL position exceed the significant threshold level divided by the number of simulations. Threshold value of the test statistic was calculated by using permutation test.

### Multiple unlinked QTLs

**Fig 1:**
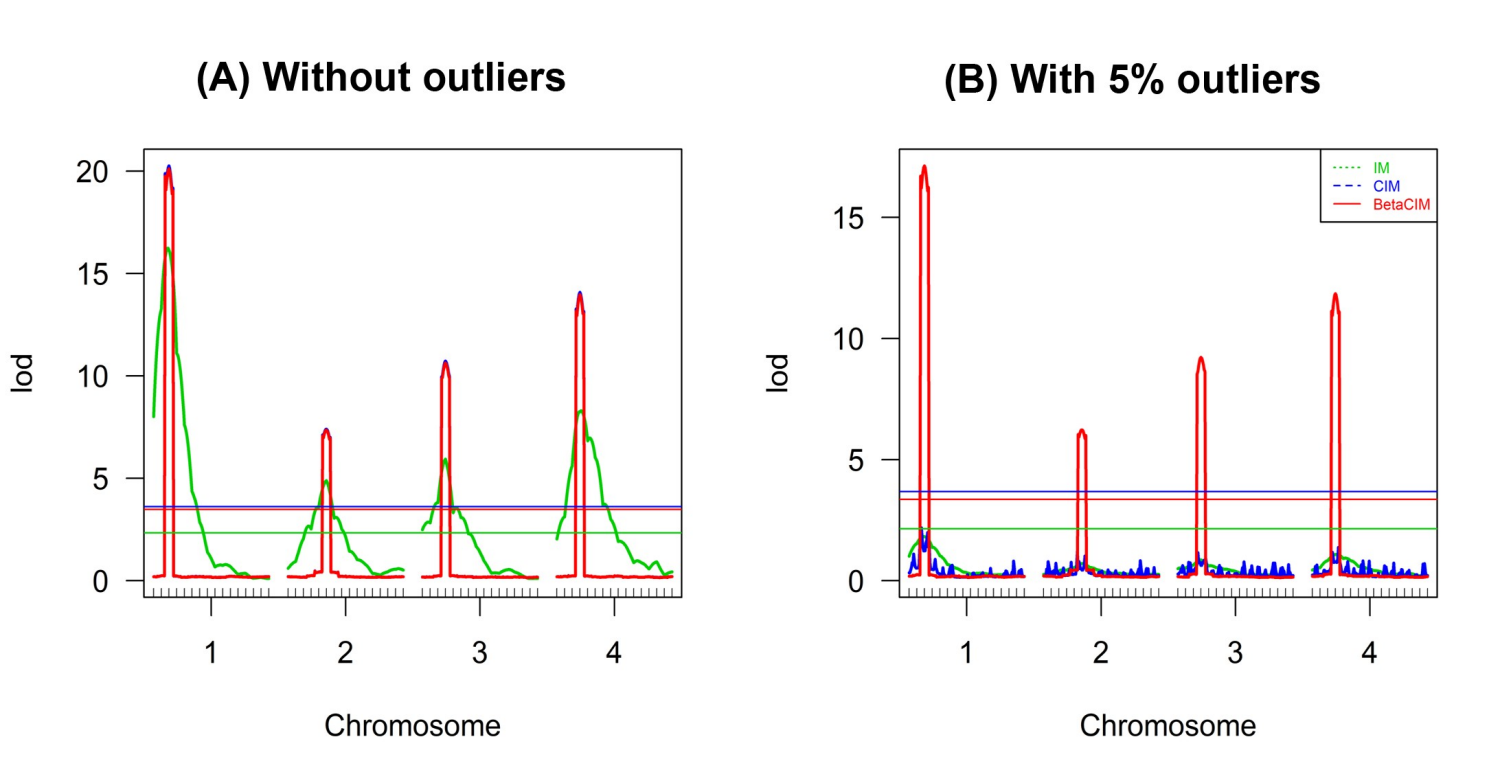
Simulations results without and with phenotype outliers in the case of multiple unlinked QTL. (A) Analysis results in absence of outliers; (B) analysis results in presence of 5% outliers. Threshold for each method were calculated using permutation test with 1000 replicates.

We simulated data for backcross population by assuming the QTLs located far enough to be linkage equilibrium to each other’s. Four QTLs situated in different chromosomes were considered for simulation study. Four chromosomes each with fifteen markers separated in 10cM intervals were simulated. Phenotypic data sets were generated by assuming some of the specific marker positions (Chromosome-Marker: C_1_M_3_, C_2_M_6_, C_3_M_4_ and C_4_M_4_) as QTLs. Variations of phenotypic data was contributed 50% by QTLs and 50% by random error. We generated 100 simulated data and analyzed by using IM, CIM, and BetaCIM approaches, and then plotted the average LOD scores (Fig 1). Sample size for each simulation was 300. Moreover, simulation was conducted with 5% contaminated phenotype data to investigate the robustness property of classical approaches and BetaCIM approaches. Same approach was used to generate contaminated data described in previous publications [12, 15], randomly selected 5% of the observations and then added random numbers.

Simulation results showed that all approaches could identify the true loci in absence of phenotypic outliers, and provided good estimates of genetic parameters with smaller standard error (Table 1).

**Table 1:** Detection power of different approach for multiple unlinked QTLs.

Marker	Parameters	IM			CIM			BetaCIM		
	*a*	Estimate	SE	Power	Estimate	SE	Power	Estimate	SE	Power
C_1_M_3_	2.12	2.16	0.23	97	2.13	0.39	100	2.13	0.39	100
C_2_M_6_	-1.23	-1.40	0.20	88	-1.19	0.38	91	-1.19	0.38	91
C_3_M_4_	-1.46	-1.48	0.19	98	-1.49	0.34	98	-1.49	0.34	98
C_4_M_4_	1.74	1.65	0.20	64	1.72	0.32	99	1.72	0.32	99
With 5% Outliers										
C_1_M_3_	2.12	2.30	0.80	27	2.22	0.84	31	2.16	0.38	100
C_2_M_6_	-1.23	-1.39	0.67	2	-1.16	0.99	6	-1.22	0.42	88
C_3_M_4_	-1.46	-1.52	0.73	8	-1.48	0.87	15	-1.49	0.34	97
C_4_M_4_	1.74	1.79	0.68	8	1.83	0.97	18	1.68	0.36	98

Analysis results for 100 simulations with multiple unlinked QTL. Marker: genetic markers; Parameters: genetic effects of QTL; Estimate: estimated value of genetic parameters; SE: standard error of the estimates; Power: detection power of the QTLs.

Although, detection powers of 3 QTL positions were similar for IM, CIM and BetaCIM approaches, for QTL C_4_M_4_ detection power of IM approach was low (power = 64%) as compared to others two methods. Therefore, in the case of multiple unlinked QTL, detection power of the QTLs is better for CIM and BetaCIM approaches as compared to IM. Moreover, in the case of 5% phenotypic outlying observations, IM and CIM approaches failed to detect true QTL positions (Fig 1B). Detection powers of these approaches were very low (8~27% for IM, and 6~31% for CIM), as well as standard errors of the parameters were very high, implying the estimated genetic effects highly varied across different simulated data sets (Table 1). Interestingly, the BetaCIM method provides consistent results, just as there is no outlying observations, identified all QTL positions with high power and provided good estimates of genetic parameters with smaller standard errors (Table 1). Therefore, BetaCIM approach significantly improved the performance over classical CIM approach in the case of multiple unlinked QTLs and presence of phenotypic outliers.

### Multiple linked QTLs

One of the crucial properties of CIM is the ability of identifying multiple linked QTLs. In this scenario, we conducted simulation with similar parameter setting as previous publication for CIM [6]. We simulated data from four chromosomes, each with 16 markers and separated in 15 10cM intervals. The traits were controlled by 10 QTLs with positions and effects given in Table 2. Among the 10 QTLs, 9 were in 1^st^ three chromosomes and 1 was in chromosome 4.

**Table 2:** Parameters and point estimates of effects with and without phenotypic outliers.

	Chr 1			Chr 2			Chr 3			Chr 4	
QTL	C_1_M_3_	C_1_M_6_	C_1_M_12_	C_2_M_2_	C_2_M_6_	C_2_M_9_	C_3_M_4_	C_3_M_8_	C_3_M_14_	C_4_M_4_	MSE
Position (cM)	20	50	110	10	50	80	30	70	130	40	
Effect	0.42	0.75	0.58	1.02	-1.23	-1.26	-0.46	1.61	0.88	0.74	
Without Outliers											
IM	0.75	0.95	0.57	0.41	-1.37	-1.55	0.26	1.37	1.07	0.69	0.124
CIM	0.45	0.85	0.61	1.00	-1.20	-1.32	-0.45	1.37	0.88	0.77	0.007
BetaCIM	0.45	0.84	0.61	1.00	-1.20	-1.32	-0.45	1.37	0.88	0.76	0.007
With 5% Outlying Observations											
IM	-0.57	0.70	1.10	1.37	-1.41	-1.54	0.55	1.33	0.69	0.28	0.283
CIM	-1.29	-1.01	1.00	2.17	-1.74	-1.10	0.01	1.40	0.55	0.26	0.841
BetaCIM	0.51	0.46	0.59	1.05	-1.26	-1.26	-0.37	1.33	0.90	0.73	0.018

Simulation results from 1 replicate with total heritability 70%. C_i_M_j_ denote the j^th^ marker of i^th^ chromosome; Position: QTL position; Effect: QTL effect.

**Fig 2:**
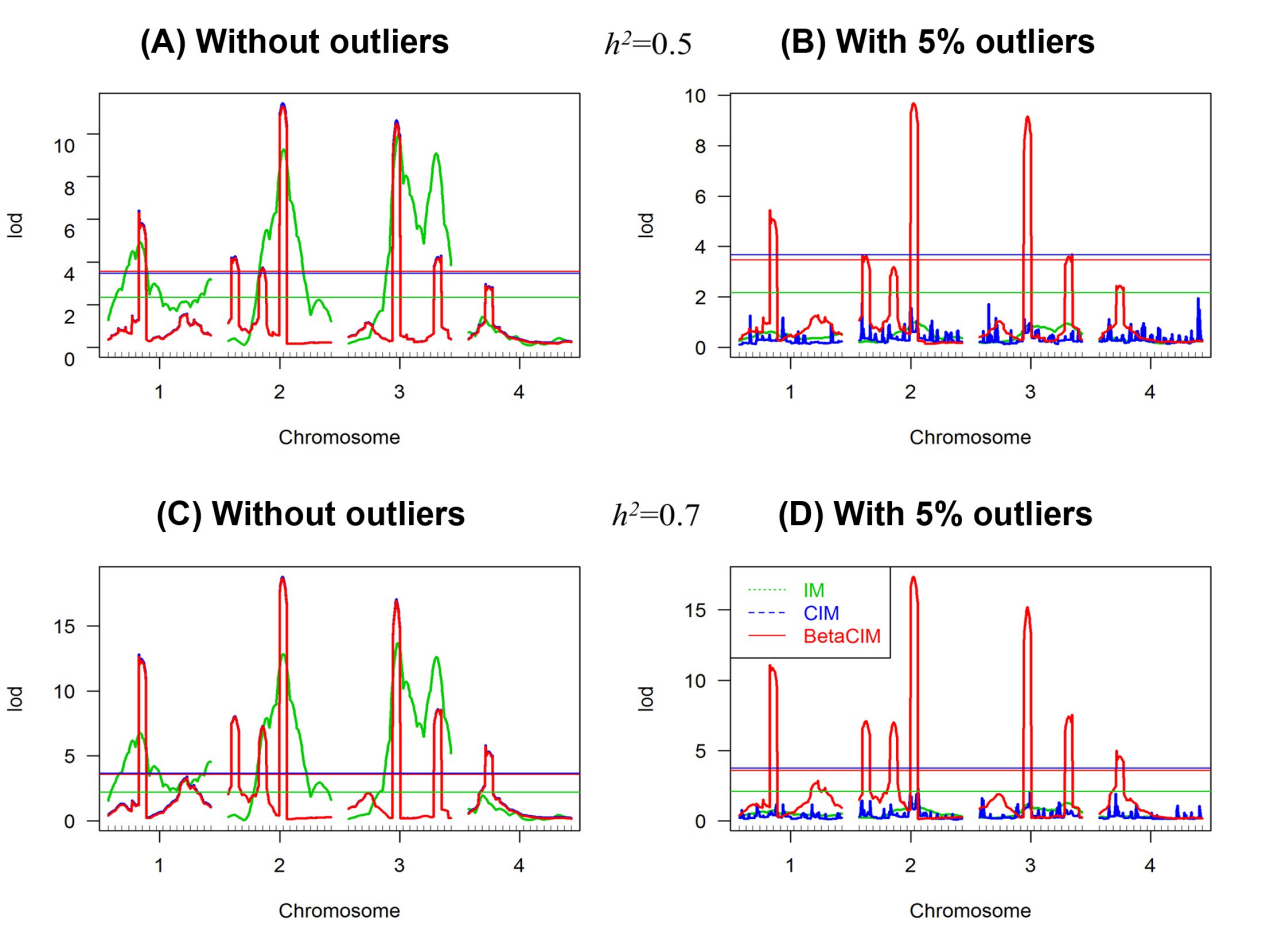
Simulation results in presence and absence of phenotypic outliers in the case of multiple linked QTLs. Total genetic heritability was set as 50% and 70% for two different sets of simulated data. 5%-contaminated data was added to phenotypes to investigate the robustness property of the approaches. (A) Analysis results in the case of 50% genetic heritability and no outlying observation; (B) analysis results in the case of 50% genetic heritability and 5% outlying observations; (C) Analysis results in the case of 70% genetic heritability and no outlying observation; (D) analysis results in the case of 70% genetic heritability and 5% outlying observations. Threshold for each method were calculated using permutation test with 1000 replicates.

Together, the QTLs account for 50% and 70% of the phenotypic variance for two different sets of simulated data. Sample size was 300. In Fig 2, we plotted the average LOD scores for 300 simulations under two different scenarios, with and without phenotypic outliers, and for two different heritability settings. Results showed that BetaCIM can provide similar LOD score profile as CIM approach in absence of phenotypic outliers (Fig 2A and 2C).

With 50% genetic heritability both approaches identified 6 QTLs out of 10 QTLs at 5% level of significance (Fig 2A). These approaches provided lower picks at others five true QTL positions. Although, with 70% genetic heritability both approaches detected one additional QTL positions, referring that increasing of genetic heritability of the phenotypic traits can increase the detection power of the approaches. IM approach detected 4 true QTL positions with 50% and 70% genetic heritability respectively. This approach provided wider picks, failed to separate multiple linked QTLs and detected several wrong QTL positions (Fig 2A and 2C). Therefore, in the case of multiple linked QTLs, CIM and BetaCIM provided better result than IM approach.

Simulation with single replicate showed that IM approach could provide larger mean sum square error (MSE = 0.124) compared to CIM and BetaCIM approaches, referring that parameter estimation of IM approach could be biased in the case of multiple linked QTL. CIM and BetaCIM approaches provided similar estimates of the genetic parameters and equal mean sum square error (MSE = 0.007), indicating the CIM and BetaCIM approaches could provide better results compared to the IM approach in the case of multiple linked QTL.

With 5% contaminated observations, CIM and IM failed to detect true QTL positions (Fig 2B and 2D). However, BetaCIM provided similar results as without outliers, detected the true QTL positions (Fig 2B and 2D). Therefore, BetaCIM is robust against phenotypic contamination, can detect true QTL positions in presence and absence of outliers. Outlying observations had large impact on parameter estimation of IM and CIM approaches. Estimation was biased upward or downward for these approaches (Table 2), and provided larger MSE. BetaCIM approach significantly improved the performance in effects estimation and identifying true QTLs in the case of outlying observations, provided smaller MSE. Simulation with *F*_2_ population provided similar results for the approaches (Text A, Figs A and B, and Table A in S1 File).

### Beta selection for QTL analysis

**Fig 3:**
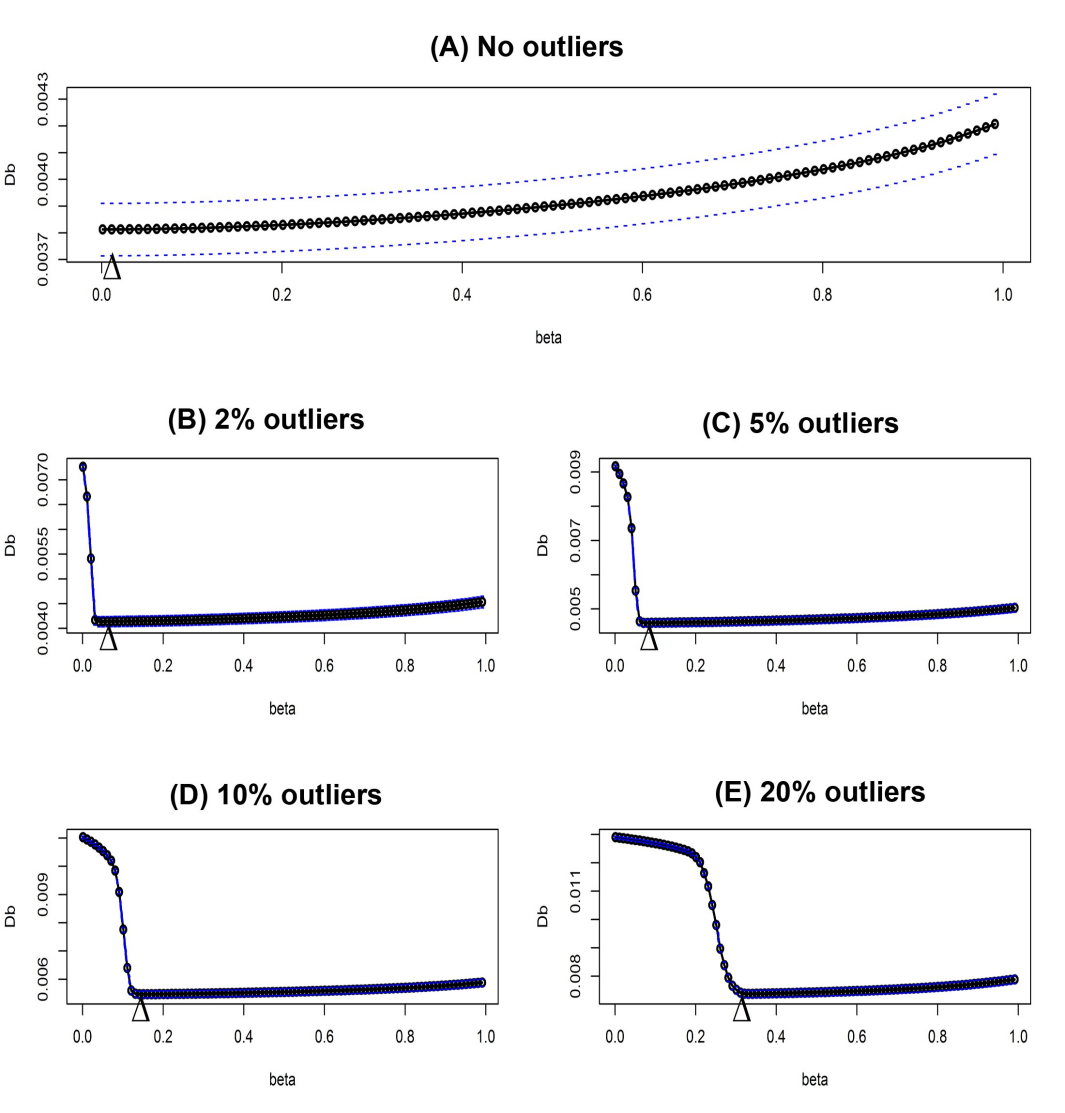
Optimum value of the tuning parameter beta with different proportion of phenotypic outliers. Triangles are locating at the median optimum value of beta. (A-E) plots for *β* selection by cross-validation in presence of 0%, 2%, 5%, 10% and 20 outliers, respectively.

In simulation study, we observed that BetaCIM approach significantly improved the analysis results in presence and absence of phenotypic outliers. Tuning parameter *β* plays key role for controlling effects of contaminated data. Value of *β* depends on proportion of contamination in phenotypic data; its value can increase with respect to increasing outlying observations. We calculated the average value of *β* with different proportion of phenotypic outliers (Fig 3).

In 100 simulations without phenotypic outliers, the median of optimum values of the tuning parameter *β* was 0.001, referring that without phenotypic outlier optimum value of *β* is very small. We observed that optimum value of the tuning parameter *β* increase with respect to increasing outlying observations. For example, in the case of 2~20% of outlying observations median of optimum values of *β* were varies to 0.041~0.291. Therefore, selecting trait specific tuning parameter *β* by using cross validation is crucial for the BetaCIM approach. Selecting the tuning parameter could be useful for real data analysis that may help in efficient estimation of QTL positions and effects. Implemented function of “BetaCIM” R-package can select trait specific optimum beta for analysis.

### Real data analysis

**Fig 4:**
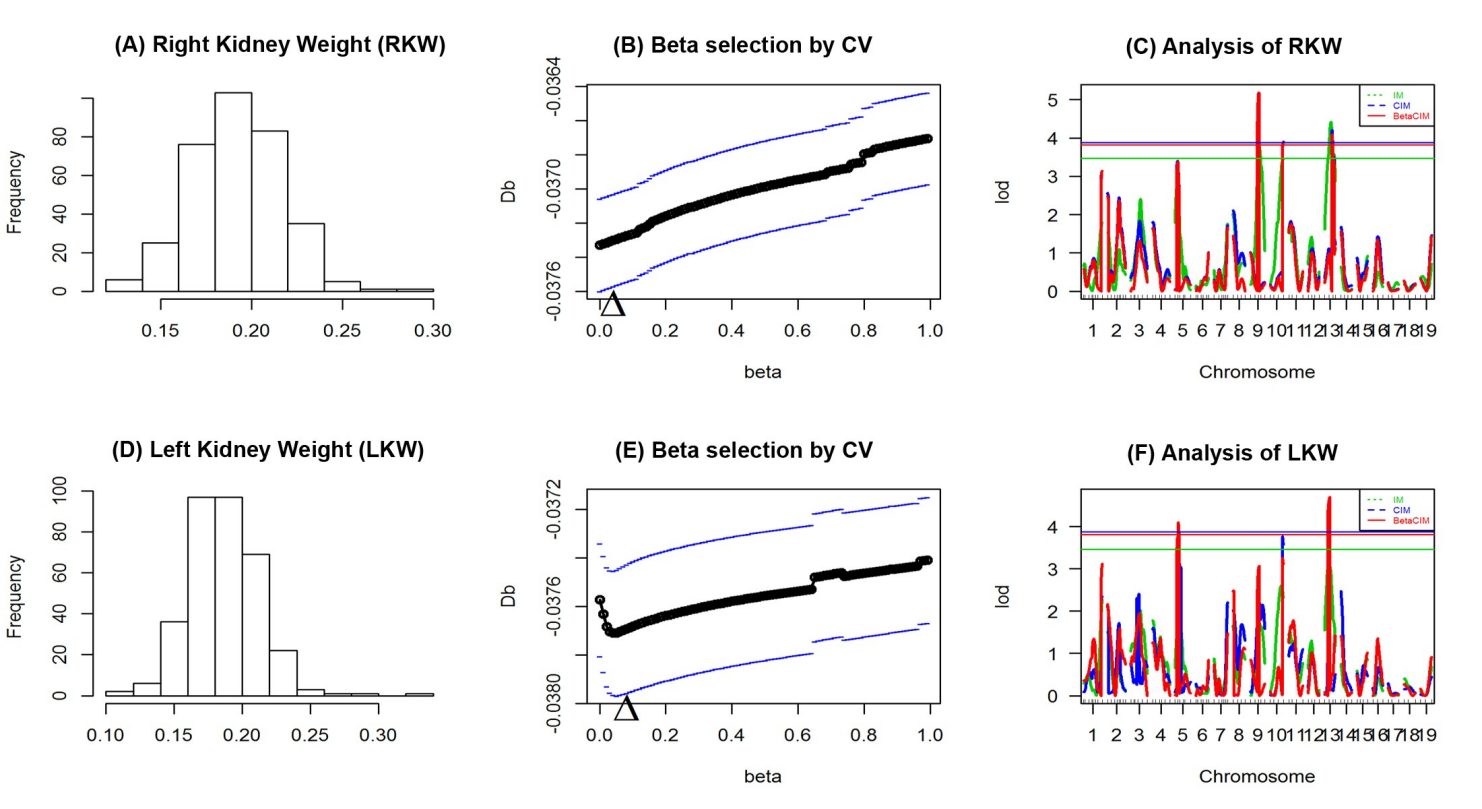
Analysis of kidney weight data of mouse intercross population (129 S1/SvlmJ× A/J). (A, D) Plots of phenotypic distributions for right and left kidney weights, respectively; (B, E) Plots for *β* selection by cross-validation from left and right kidney weights, respectively. (C, F) LOD score profiles for left and right kidney weights, respectively. Threshold for each method were calculated using permutation test with 1000 replicates.

Data from an experiment on multiple traits in the mouse was downloaded from mouse phenome database (https://phenome.jax.org/projects/Feng1). The data was for an intercross between 129S1/SvlmJ and A/J inbred mouse strains. There were several phenotypic traits scored in the cross. We analyzed left and right kidney weight of mouse to identify the QTLs underlying these traits. There were total 336 intercross individuals, aged 8 weeks, and typed at 91 markers. For more details about the data see the related publication [23]. IM, CIM and BetaCIM approaches were used for analyses (Fig 4).

From Fig 4A, we observed that the right kidney weight (RKW) data symmetrically distributed and there had no extreme observations. None of the phenotypic observation was larger than Q_3_+3*IQR or smaller than Q_1_-3*IQR, where Q_1_, Q_3_ and IQR are first quartile, 3^rd^ quartile, and inter quartile range of RKW data. In this case, optimum value of beta was 0.001 that also indicated there had no contaminated observations in the data set. Analysis with CIM and BetaCIM approaches provided similar LOD score profile and identified 3 QTL positions at 5% level of significance (Fig 4C). Highest picks of the identified QTL positions by using CIM and BetaCIM approaches were in 42.14 cM of chromosome 9 (LOD = 5.18, LOD_*β*_ = 5.17) with nearby marker rs3676158 (position = 40.876 cM, LOD = 5.16, LOD_*β*_ = 5.15); in 67.941 cM of chromosome 10 at the marker rs3674646 (LOD = 3.90, LOD_*β*_ = 3.89); and in 45.055 cM of chromosome 13 at the marker rs3716022 (LOD = 3.53, LOD_*β*_ = 3.42). IM approach also identified three QTL positions; the highest significant picks were 45.132 cM of chromosome 9 (LOD = 3.79) with nearby marker rs3676158 (LOD = 3.67), in 67.941 cM of chromosome 10 at the marker rs3674646 (LOD = 3.46); and 35.299 cM of chromosome 13 within the marker interval rs3676930 (position 24.6737 cM) and rs3716022 (position 45.055 cM). The candidate markers rs3676158 and rs3674646 are the intron variants of genes *Unc13c*, and *Grip1* respectively, however gene information of another variant rs3716022 is unknown. *Unc13c* is responsible for an additional step of molecular and/or positional "superpriming" that substantially increases the efficacy of Ca(2+)-triggered release [24]. Its play a role in vesicle maturation during exocytosis as a target of the diacylglycerol second messenger pathway, and may be involved in the regulation of synaptic transmission at parallel fiber (http://www.uniprot.org/uniprot/Q8K0T7). *Grip1* play a role as a localized scaffold for the assembly of a multiprotein signaling complex and as mediator of the trafficking of its binding partners at specific subcellular location in neurons (http://www.uniprot.org/uniprot/Q925T6).

We analyzed the left kidney weight (LKW) data of the same population. In this case, there was evidence of one outlying phenotypic observation (phenotypic value of one individual observation was larger than Q_3_+3*IQR, where Q_3_ is the third quartile and IQR is interquartile range of LKW data). Optimum value of the tuning parameter *β* was larger than before (opt *β* = 0.041), indicated there had some contaminated observations in the data set. For LKW, IM and CIM approaches identified none of the QTL positions at 5% level of significance (Fig 4F). However, BetaCIM approach identified 2 QTL positions, which are 12.43~21.43 cM of chromosome 5 and 20.30~29.30 cM of chromosome 13. LOD scores for CIM and IM approaches at the QTL positions were high but not significant at 5% level of significance (Fig 4F). These two QTLs hold the markers rs3023765 (position = 16.550 cM, LOD_*β*_ = 3.78) and rs3676930 (position = 24.6737 cM, LOD_*β*_ = 4.54), which are the variant of the genes *Otof* and *A330033J07Rik*. Mutations in human orthologous of mice *Otof* gene are a cause of neurosensory nonsyndromic recessive deafness, hearing loss [25]. Individual with moderate chronic kidney disease (CKD) have a higher prevalence of hearing loss than those of the same age without CKD (https://www.kidney.org/news/ekidney/november10/HearingLoss_November10). Mice lacking *Otof* display hearing loss. It expressed in the cochlear IHC, vestibular type I sensory hair cells, eye, heart, skeletal muscle, liver, kidney, lung and testis (http://www.uniprot.org/uniprot/Q9ESF1). Another novel gene *A330033J07Rik* also expressed in kidney (https://www.ncbi.nlm.nih.gov/geoprofiles/7902881). Therefore, the candidate genes may have relevant function for kidney weight.

## Discussions

This paper discusses the robustification of CIM algorithm for identification of both linked and unlinked QTLs by maximizing *β*-likelihood function using the EM like algorithm. The value of the tuning parameter *β* plays a key role on the performance of the BetaCIM method. An optimum value for the tuning parameter *β* can be selected by using *k*-fold cross validation. Simulation studies showed that the value of tuning parameter *β* increase with respect of increasing proportion of outlying observations (Fig 3). Therefore, optimum value of the tuning parameter depends on data contamination rate, could vary across real data sets. We implemented the cross-validation procedure of selecting trait specific optimum *β* in BetaCIM R-package that could help to select trait specific value of the tuning parameter. In simulations, we observed that the BetaCIM approach significantly improved the performance over IM and CIM approaches in presence of outliers; otherwise, it keeps equal performance with CIM. This approach can identify all the QTL positions that can be detected by using CIM in absence of phenotypic outliers. And in presence of phenotypic outliers only BetaCIM approach can provide consistent results (Figs 1 and 2). CIM and IM approaches could fail to detect true QTL positions in presence of phenotypic outliers.

Again, simulation with 100 replicates, we observe that BetaCIM approach can provide reliable estimates of genetic parameters in presence and absence of phenotypic outliers (Table 1). The CIM and BetaCIM can provide smaller mean sum square error (MSE) compared to the IM approach in absence of phenotypic contamination (Table 2). Interestingly, in presence of phenotypic contamination MSE of CIM approach can be larger than IM approach, might due to biased estimations of the effects of background genetic makers that use as cofactor. IM approach does not use the background markers as cofactor. Therefore, genetic parameter estimation by using CIM approach is more sensitive to outliers compared to IM approach. However, in presence of phenotypic outliers BetaCIM provided smaller MSE compared to IM and CIM approaches, thus this approach overcomes the deficiency of CIM approach. Again, QTL detection powers of the BetaCIM approach are similar in absence and presence of phenotypic outliers, whereas detection power was very small for CIM and IM approaches in presence of phenotypic outliers (Table 1). In real situations, phenotypic data might be contaminated by different environmental exposures [11], as well as may contain some measurement errors. A robust approach can provide reliable results in real situations.

We analyzed two real data sets: right kidney weight (RKW) and left kidney weight (LKW) of mouse intercross population [23]. In real data analysis, trait specific optimum *β* were equal to 0.001 and 0.041 for two different datasets, indicating the presence of some unusual observations in the LKW data set (Fig 4B and 4E). After checking the phenotypic observations, we observed that there was evidence of outlying observation in LKW, but not in RKW. CIM and BetaCIM approaches provided similar LOD score profile for RKW, but different for LKW. The CIM and IM approach failed to detect the QTL positions for LKW, might due to presence of phenotypic contamination, whereas BetaCIM approach identified two QTL positions at 5% level of significance. Identified candidate genes of these QTL regions were expressed in Kidney. Simulation and real data analysis showed that BetaCIM approach could be useful to robustly identify the QTL positions and unbiased estimate of genetic parameters of experimental populations.

## Supporting information

S1 File Includes Text A, Table A, Fig A, and Fig 2. **Text A in S1 File. Simulations with Intercross Population**.**Table A in S1 File. Detection power of different approaches for multiple linked QTLs in the case of F2 population.** Results were calculated from 500 simulations. IM: Interval Mapping approach; CIM: Composite Interval Mapping approach; BetaCIM: Beta likelihood based composite interval mapping approach.**Fig A in S1 File. Results from 100 simulations for F2 population in the case of unlinked QTLs.** (A) without phenotypic outliers, and (B) with 5% phenotypic outliers. Threshold for each method were calculated using permutation test with 1000 replicates.**Fig B in S1 File.** Results from 100 simulations for F2 population in the case of multiple linked QTLs. (A) without phenotypic outliers, and (B) with 5% phenotypic outliers. Threshold for each method were calculated using permutation test with 1000 replicates.
(DOC)
