# Supplementary material for: β-composite Interval Mapping for robust QTL analysis
Source: PLoS One. 2018 Dec 3;13(12):e0208234. doi: 10.1371/journal.pone.0208234 (PMC6277098; doi:10.1371/journal.pone.0208234)
Supplement: S1 File — Text A in S1 File. Simulations with Intercross Population.Table A in S1 File. Detection power of different approaches for multiple linked QTLs in the case of F2 population. Results were calculated from 500 simulations. IM: Interval Mapping approach; CIM: Composite Interval Mapping approach; BetaCIM: Beta likelihood based composite interval mapping approach.Fig A in S1 File. Results from 100 simulations for F2 population in the case of unlinked QTLs. (A) without phenotypic outliers, and (B) with 5% phenotypic outliers. Threshold for each method were calculated using permutation test with 1000 replicates.Fig B in S1 File. Results from 100 simulations for F2 population in the case of multiple linked QTLs. (A) without phenotypic outliers, and (B) with 5% phenotypic outliers. Threshold for each method were calculated using permutation test with 1000 replicates. (DOC) [file pone.0208234.s001.doc]

*β***-Composite Interval Mapping for Robust QTL Analysis**

Md. Mamun Monir^1,2^, Mita Khatun^2^, and Md. Nurul Haque Mollah^1*^

^1^ Department of Statistics, University of Rajshahi, Rajshahi-6205, Bangladesh.

^2^ Institute of Bioinformatics, Zhejiang University, Hangzhou, 310058, China.

*Corresponding Author:

Email: [mnhmollah@yahoo.co.in](mailto:mnhmollah@yahoo.co.in)

**Text A. Simulations with Intercross Population**

**Multiple unlinked QTLs.** We simulated data by assuming the QTLs located far enough to be linkage equilibrium to each other’s. Total three QTLs were considered for simulation study, whereas they situated in three different chromosomes. Four chromosomes each with fifteen markers separated in 10cM intervals were simulated. Phenotypic data sets were generated by assuming some of the specific marker positions (Chromosome-Marker: C_1_M_2_, C_2_M_3_, and C_3_M_7_) as QTLs. Variations of phenotypic data was contributed 40% by QTLs and 60% by random error. In this setting, the performance of mapping approaches was investigated. We generated 100 simulated data and analyzed by using interval mapping, composite interval mapping, and proposed method, and then plotted the average LOD scores (Fig A in S1 file). Again, 5% phenotype data was contaminated to observe the robustness property of classical approaches and proposed approach (Fig A in S1 file).

Simulation study showed that the classical approaches and proposed approach can identify true QTL positions in absence of phenotypic outliers (Fig A in S1 file). However, QTL peaks were relatively wider for interval mapping approach compare to the composite interval mapping and proposed approach. In presence of 5% contaminated data, we observed that only proposed method successfully identified 3 QTLs. Interval mapping approach and composite interval mapping approach failed to detect true QTLs and produced several random peaks over the genome (Fig A in S1 file). This simulation suggesting that the proposed approach significantly improved the power of detecting true QTLs in presence of phenotypic outliers.

**Multiple linked QTLs.** One of the crucial properties of composite interval mapping is the ability of identifying multiple linked QTLs. We simulated phenotypic data by considering multiple linked QTL to observe the performance of proposed and classical approaches in presence and absence of phenotypic outliers. Four chromosomes each with 16 markers separated in 10cM intervals were simulated. Phenotypic data sets were generated by assuming some of the specific marker positions (Chromosome-Marker: C_1_M_2_, C_1_M_5_, C_2_M_3_, C_2_M_8_, C_2_M_13_, C_3_M_7_) as QTLs. The QTLs account for 60% of the phenotypic variance and sample size was 250. To observe the performance of the methodologies in presence of phenotypic contamination, we randomly allocated 5% of phenotype outliers. Analyses results were plotted in Fig B in S1 file.

In this case CIM and proposed approach provides similar results and identified 6 QTL positions (Fig B in S1 file). LOD score for another locus was high but smaller than the critical value at 5% level of significance. IM approach also identified 6 QTL positions, but provided additional picks close to true QTL positions. Again, estimated QTL regions were wider for IM then the CIM and proposed approach. That illustrates the efficiency of CIM approach and proposed approach over IM approach to identify the true QTL positions in the case where multiple nearby QTLs have control phenotypic traits. In the case of 5% outlying observations IM approach failed to detect all the QTLs. CIM approach identified only one QTL. However, proposed approach identified all the QTLs, suggesting this approach can identify the true QTL positions with and without phenotypic outliers in the case of multiple unlinked QTLs.

**Table A. Detection power of different approaches for multiple linked QTLs in the case of *F*_2_ population**

| QTL | Effect | | *h*^2^=40% | | | | | |
| --- | --- | --- | --- | --- | --- | --- | --- | --- |
|  |  |  | Power (no outliers) | | | Power (5% outliers) | | |
|  | *a* | *d* | IM | CIM | BetaCIM | IM | CIM | BetaCIM |
| C_1_M_2_ | 0.42 | 0.20 | 72.4 | 89.8 | 88.8 | 11.7 | 13.4 | 85.8 |
| C_1_M_5_ | 0.30 | 0.30 | 85.0 | 88.4 | 88.0 | 11.9 | 12.0 | 84.4 |
| C_2_M_3_ | 0.20 | 0.30 | 3.8 | 59.2 | 56.2 | 2.9 | 4.0 | 48.0 |
| C_2_M_8_ | 0.25 | 0.40 | 99.7 | 90.2 | 90.2 | 15.5 | 8.6 | 90.6 |
| C_2_M_13_ | 0.30 | 0.24 | 99.7 | 88.2 | 87.2 | 1.6 | 6.0 | 83.4 |
| C_3_M_7_ | 0.20 | 0.23 | 3.3 | 59.4 | 56.2 | 1.2 | 2.4 | 49.8 |


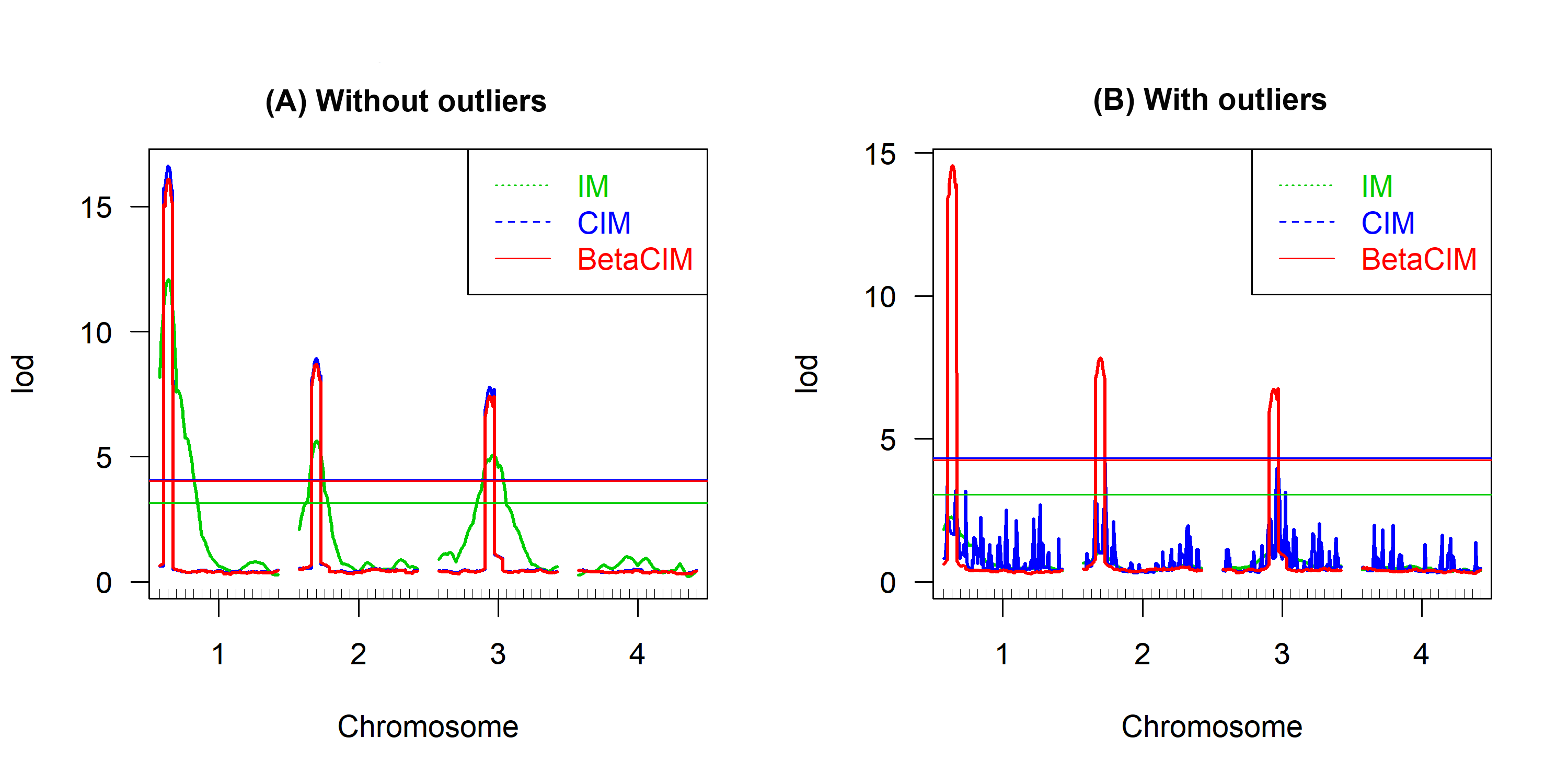


**Fig A. Results from 100 simulations for *F*_2_ population in the case of unlinked QTLs.**


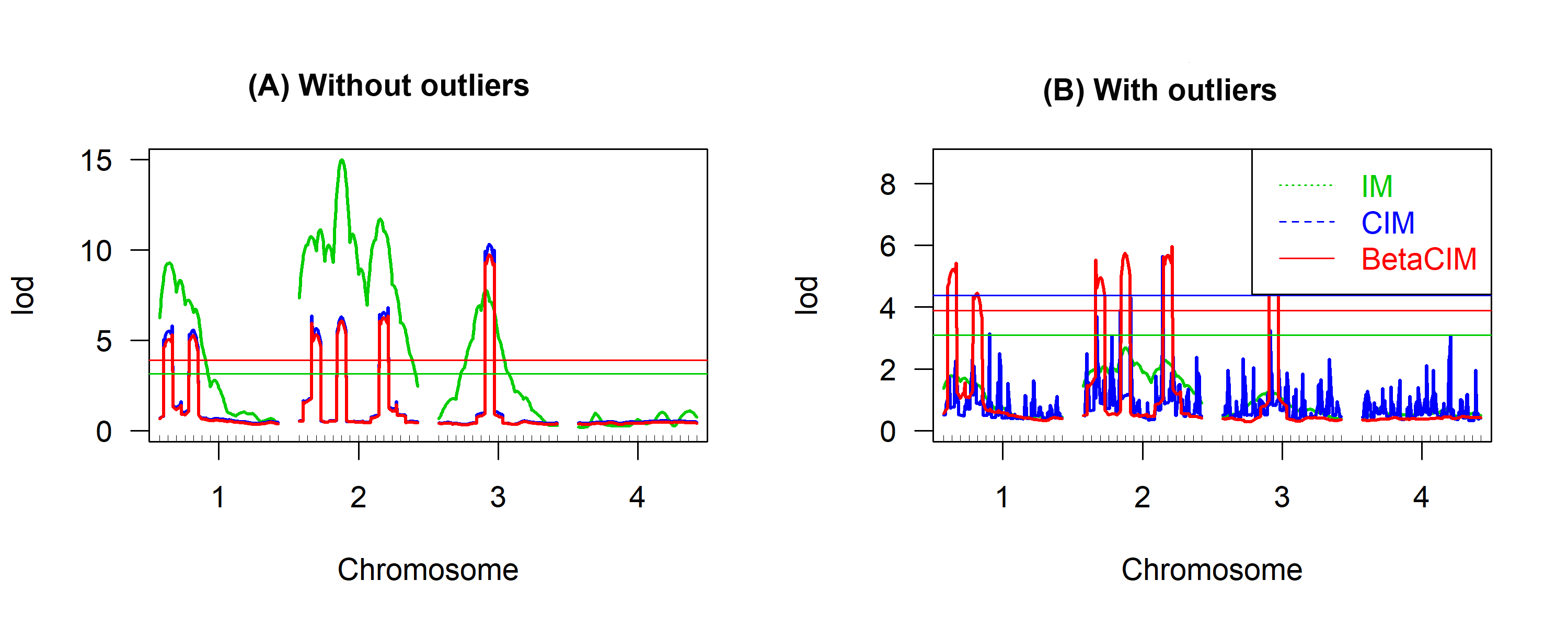


**Fig B. Results from 100 simulations for *F*_2_ population in the case of multiple linked QTLs.**
